# Enhanced HIF2α expression during human trophoblast differentiation into syncytiotrophoblast suppresses transcription of placental growth factor

**DOI:** 10.1038/s41598-017-12685-w

**Published:** 2017-09-29

**Authors:** Tatsuya Fujii, Takeshi Nagamatsu, Kazuki Morita, Danny J. Schust, Takayuki Iriyama, Atsushi Komatsu, Yutaka Osuga, Tomoyuki Fujii

**Affiliations:** 1 Department of Obstetrics and Gynecology, Faculty of Medicine, The University of Tokyo, Tokyo, Japan; 2 Department of Obstetrics, Gynecology and Women’s Health, University of Missouri, Columbia, MO USA

## Abstract

Placental growth factor (PlGF), abundantly produced from trophoblasts is involved in placental angiogenesis. The regulatory mechanism of its expression is poorly understood. Hypoxia inducible factors (HIFs) are centrally involved in the modulation of cellular function in response to low oxygen conditions. This study aimed to clarify HIF1α and HIF2α expression patterns during cytotrophoblast differentiation into syncytiotrophoblast and the impact of any changes on PlGF expression. HIF proteins were induced remarkably under low oxygen condition (2%). HIF1α expression decreased and HIF2α expression increased when syncytialization of cultured cytotrophoblasts is progressed. Those expression changes of HIF proteins in the process of *in-vitro* syncytialization was congruent with the immunohistochemical findings in preeclamptic placenta as well as uncomplicated placenta. Low oxygen condition was also associated with reduced PlGF production in syncytializing primary cells and BeWo choriocarcinoma cells. Small interfering RNA-mediated HIF2α knockdown in BeWo cells abrogated hypoxia-associated decreases in PlGF secretion; HIF1α silencing had no significant effect on PlGF secretion. In summary, HIF2α, rather than HIF1α, is most affected by reduced oxygen level during syncytialization and increases in HIF2α trigger a reduction of PlGF production. Our findings suggest new and important connections between HIF proteins and PlGF pathways in the regulation of placental angiogenesis.

## Introduction

Drastic alterations in local oxygen concentration accompany placental development. Oxygen concentrations in the inter-villous space at 8 weeks of gestation is estimated to be about 3 to 5%; this increases to about 8 to 10% after the completion of uterine spiral artery remodeling^1^. Appropriate changes in trophoblast cellular function in response to these gestational-age specific alterations in placental oxygenation are central to healthy placentation^2^.

Insufficient oxygen supply and ischemia-reoxygenation injury in dysfunctional placenta are commonly implicated in the development of preeclampsia (PE)^3^. More specifically, inadequate establishment of spiral artery remodeling during early placentation results in inadequate provision of maternal blood to the intervillous space of the human placenta and failure to satisfy fetal oxygen requirements as pregnancy progresses^4^. In the preeclamptic placenta, intermittent blood flow accumulates oxidative stress in the syncytiotrophoblast (STB), existing at fetomaternal interface^5^. The cellular stress might lead to the production of STB-derived pathogenic factors that cause systemic symptoms in the mother^6^.

The transcription factors hypoxia inducible factors (HIFs) 1α and 2α are central to the process of adaptation to hypoxic conditions by controlling the expression of genes involved in diverse biological processes such as angiogenesis, vascular tone, glycolysis, mitochondrial function, cell growth and survival^7^. As their names indicate, HIF protein functions rely on the local oxygen concentrations. HIFs are rapidly degraded in a post-translational manner under normal oxygen condition and thereby lose their functional activities. Under hypoxic/ischemic conditions, HIF proteins are stabilized and enhance the expression of target genes^8^. Several biological characteristics of HIF1α and HIF2α differ. HIF1α expression is relatively ubiquitous and appears to be closely linked to the maintenance of homeostasis. HIF2α expression, however, is restricted to specific tissues and cell types^9^. As recently described in macrophages, the two HIF proteins can differ in their regulation of the vascular endothelial growth factor (VEGF) system. Here, HIF1α promoted VEGF expression, whereas HIF2α promoted soluble fms-like tyrosine kinase 1 (sFlt-1) expression under the presence of cytokine granulocyte-macrophage colony-stimulating factor^10^. Past studies have shown HIF proteins are essential in successful embryo implantation and placentation. Gene deletion of HIF1α or HIF2α resulted in impaired trophoblast differentiation, placental malformation and fetal death in mice^11^. The gene product of the von Hippel-Lindau (VHL) gene is essential for degradation of HIF proteins^12^. Uncontrolled enhancement of HIF proteins by VHL gene deletion causes deranged vasculogenesis in the murine placenta^13^. These observations suggest HIF proteins are critical for trophoblast differentiation and the regulation of placental vascular formation. In the human placenta, the significance of HIF proteins in trophoblast differentiation and placental angiogenesis has not been fully elucidated.

The VEGF system is central to the regulation of the angiogenic processes. In this system, vascular development and function are regulated by a balance between proangiogenic factors, represented by VEGF and placental growth factor (PlGF), and anti-angiogenic factors such as sFlt-1. Through its binding to VEGF and PlGF, sFlt-1 inhibits the biological actions of those proangiogenic factors^14^. Gestational stage specific regulation of proangiogenic and anti-angiogenic balance is critical especially in the early stage of pregnancy. Dominant action of anti-angiogenic factors is estimated to be vital for proper implantation and embryo development, while the shift to proangiogenic activities are required in the later stage of placental vasculature development^15^. Ample production of proangiogenic and anti-angiogenic members of the VEGF family by trophoblast cells aids in the establishment of appropriate fetomaternal circulation. The molecular mechanisms to regulate those angiogenic molecules in trophoblast cells are not fully elucidated.

In this study, we aimed to investigate the expression patterns of HIF1α and HIF2α in human trophoblast cells during differentiation from cytotrophobalsts (CTBs) to STB and the impact of these HIF proteins on PlGF expression.

## Results

### *In-vitro* syncytialization of isolated cytotrophoblasts

**Figure 1:**
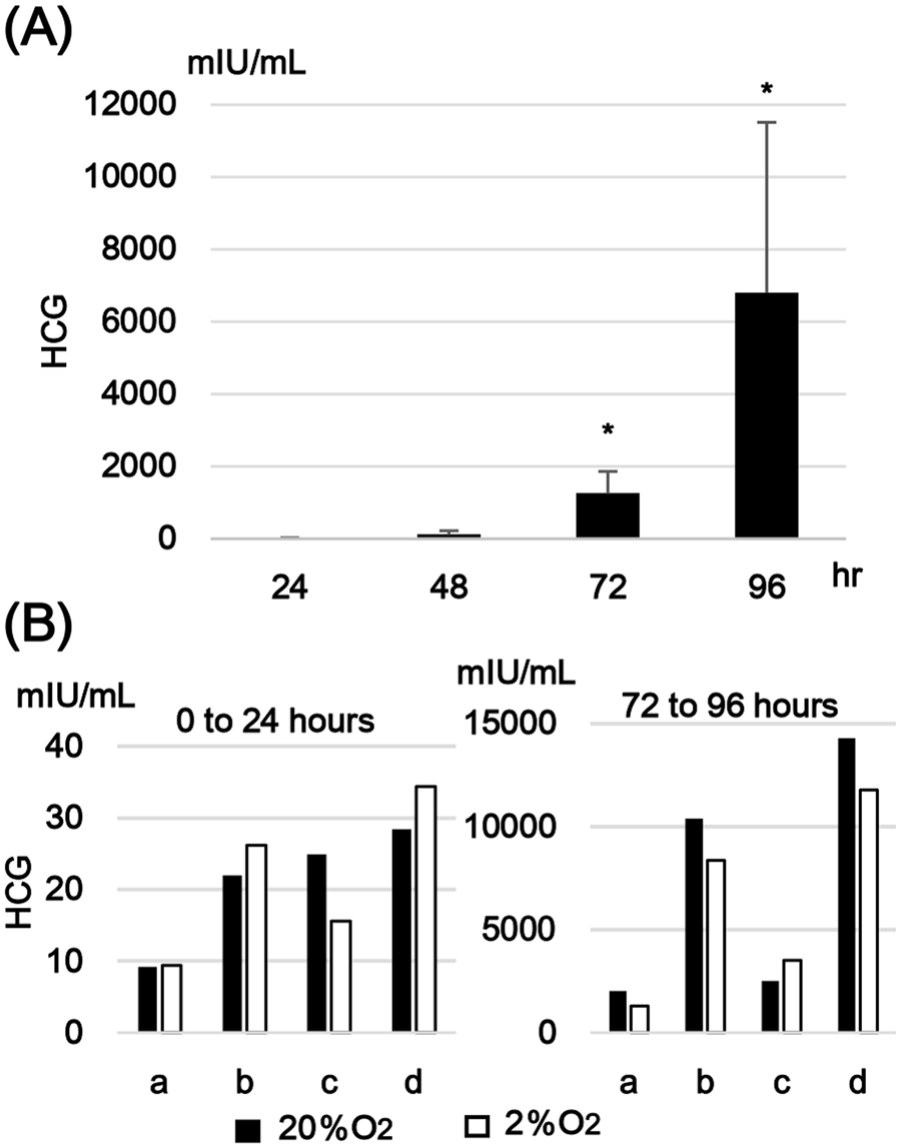
HCG secretion from cultured trophoblast cells. (**A**) Transition of HCG secretion in the process of *in-vitro* syncytialization. Isolated cytotrophoblasts were seeded on a culture plate and the culture supernatants were collected and the medium was replenished every 24 hours. HCG concentration in the culture supernatants were measured using chemiluminescence enzyme immunoassay. The shown data were derived from independent six experiments using cytotrophoblasts isolated from different placental tissues. *p < 0.05 compared to the results of 24 hours cultured supernatants. (**B**) Impact of hypoxia on HCG secretion. Freshly Isolated cytotrophoblasts (CT) and syncytiotrophoblast (ST) obtained by spontaneous differentiation of the cytotrophoblasts at 72 hours in culture were prepared. The CT and the ST were incubated under distinctive oxygen environments, 20% O_2_ or 2% O_2_ conditions for 24 hours. HCG concentrations in the culture supernatants were determined. The experiments were repeated four times using cytotrophoblasts isolated from four different placentas, naming a, b, c and d.

After seeding the isolated cytotrophoblasts, spontaneous cell fusion occurred similarly to syncytialization *in vivo*. Morphological change into multinuclear cell clusters was evident at 72 hours. By 96 hours in culture, the culture wells were covered by a monolayer of syncytialized trophoblast. It is known that syncytiotrophoblast is characterized by abundant secretion of human chorionic gonadotropin (HCG)^16^. After seeding, culture supernatants were collected and new medium was replenished every 24 hours up to 96 hours in culture. HCG concentrations in the collected supernatants were measured using ELISA to confirm that the observed morphological changes were accompanied by secretory properties corresponding to syncytiotrophoblast. HCG secretion increased rapidly over time, particularly after 72 hours (n = 5–6, respectively, Fig. 1A). The acquisition of HCG secretion capacity suggested that the stages of cell differentiation in our cultured trophoblast model corresponded to cytotrophoblasts during the period of 0 to 24 hours in culture and syncytiotrophoblast during the period of 72 to 96 hours in culture.

We next examined the impact of the oxygen environment on our cultured trophoblasts. Cultures were exposed to 2% O_2_ for 24 hours at distinct periods after seeding, from 0 to 24 hours (to model cytotrophoblast cells prior to fusion) and from 72 to 96 hours (to model syncytiotrophoblast after cell fusion). No remarkable alteration in the cell morphology and the syncytialization status was observed after 2% O_2_ exposure in compared with the cells cultured in ambient air. The culture medium was replenished at the beginning of low oxygen exposure and was collected at the end of the exposure. HCG secretion during 2% O_2_ exposure periods was compared with that from the cultured trophoblasts under 20% O_2_ condition (n = 4, respectively). There were no significant differences in HCG secretion levels between 2% O_2_ and 20% O_2_ conditions in either of the exposure period groups (Fig. 1B). These findings suggested that O_2_ concentration did not affect HCG production capacity in cytotrophoblasts or syncytiotrophoblast.

### Alternations in HIF1α and HIF2α expression during the process of syncytialization

**Figure 2:**
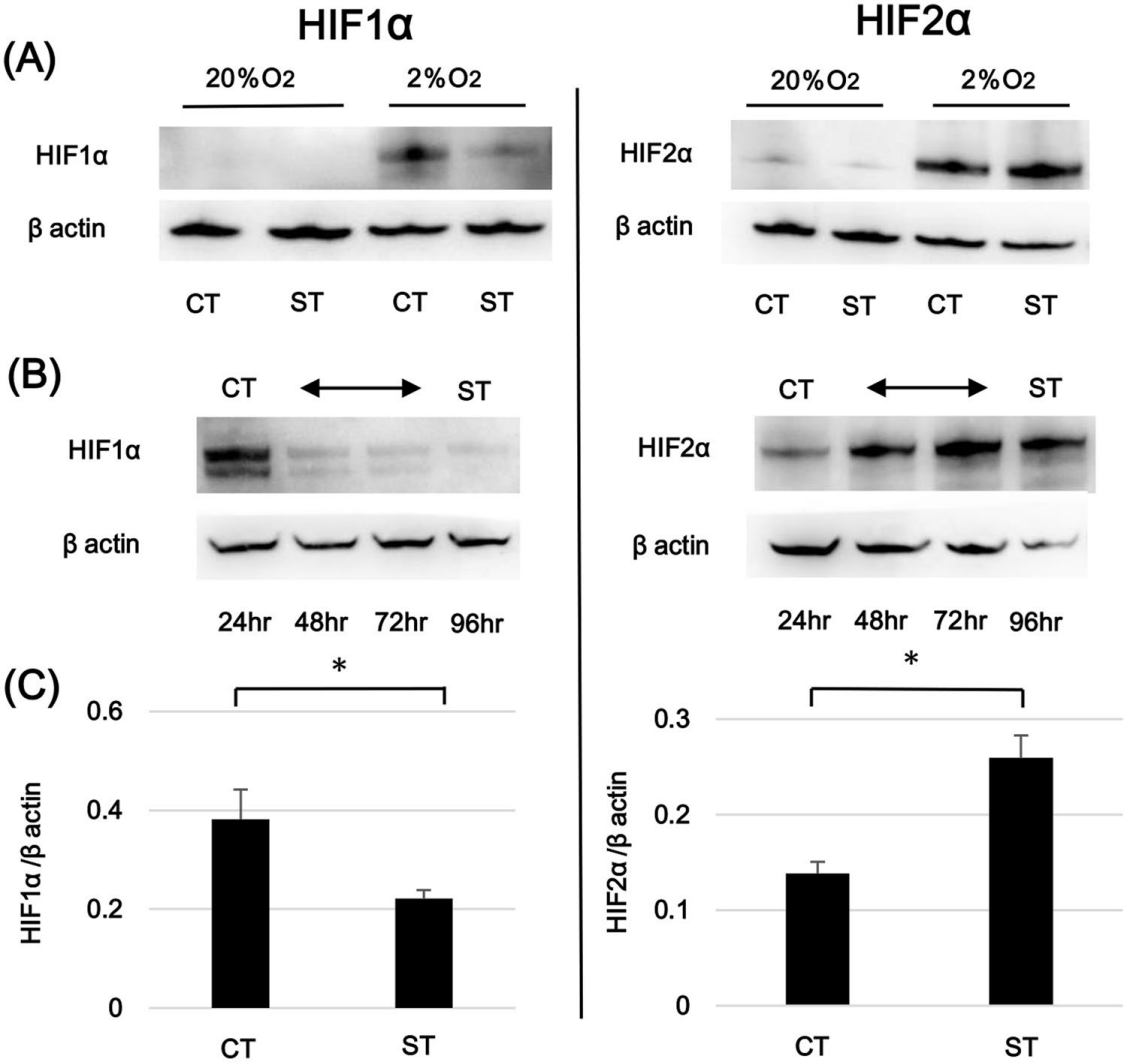
Alteration of HIFα isoform expression pattern in the process of syncytialization. (**A**) Comparison of HIFα proteins between 20% O_2_ and 2% O_2_ conditions. Freshly Isolated cytotrophoblasts (CT) and syncytiotrophoblast (ST) obtained by spontaneous differentiation of the cytotrophoblasts at 72 hours in culture were prepared. The expressions of HIF1α and HIF2α proteins in CT and ST were evaluated by western blotting analysis after 24 hours exposure to different oxygen environments. β actin specific bands were detected for normalization of protein concentration variations among the samples. A representative data of three independent experiments is shown. Cropped gels/blots around 100 kDa for HIFs and 45 kDa for β actin are displayed. (**B**) Time sequential analysis of HIFα proteins under 2% O_2_ condition. Cytotrophoblasts freshly isolated from term human placentas were cultured up to 96 hours. The cells were incubated at 2% O_2_ condition for the final 24 hours and the alteration of HIF1α and HIF2α expressions in syncytialization were analyzed at the different time points in culture, 24 hours, 48 hours, 72 hours and 96 hours. In the same manner as (**A**), the detections of HIFα proteins and β actin were conducted by Western blotting. Cropped gels/blots around 100 kDa for HIFs and 45 kDa for β actin are displayed. (**C**) The shift of HIFα gene expressions in the process of syncytialization. CT and ST were prepared similarly to (**A**). After incubation under 2% O2 condition, the mRNA expression levels for HIF1α and HIF2α were evaluated by real-time PCR in CT and ST. The shown data are based on eight experiments using trophoblast cells from different placentas. *p < 0.05.

HIF1α and HIF2α expression patterns during trophoblast differentiation were analyzed. Isolated trophoblasts were incubated for 0, 24, 48 and 72 hours in ambient oxygen conditions (20% O_2_) and were further incubated under 2% O_2_ or 20% O_2_ conditions for the final 24 hours. HIF1α and HIF2α proteins expression levels were consistently low under 20% O_2_ both in cytotrophoblast cells at 24 hours and syncytiotrophoblast at 96 hours of culture. HIF1α and HIF2α protein levels were increased in all cultures grown under hypoxic conditions (2% O_2)_ when compared to those grown under ambient oxygen conditions (Fig. 2A). Additional analysis revealed that syncytiotrophoblast HIF1α levels at 96 hours after 24 hours exposure to 2% O_2_ conditions were lower than those from cytotrophoblast cells exposed to hypoxia for 24 hours. In contrast, HIF2α levels were increased in syncytiotrophoblast compared to cytotrophoblast after 24 hours of exposure to hypoxic conditions. To further delineate the relationship between trophoblast differentiation stage and HIFα isoform expression, we examined alterations in HIF1α and HIF2α protein levels across the entirety of the 96 hours of culture. The final 24 hours in culture were in the presence of 2% O_2_. HIF1α protein induction under 2% O_2_ condition progressively diminished over time as the amount of syncytialization increased, with the largest decrease noted between 24 and 48 hours. In contrast, HIF2α protein induction in response to hypoxia increased over time as syncytialization progressed (Fig. 2B). We also assessed changes in transcription of HIF1α and HIF2α in this culture model. As predicted by protein assessments, the hypoxia-induced increase in HIF1α mRNA expression in syncytiotrophoblast was significantly lower than that in cytotrophoblast stage cells after 24 hours exposure to 2% O_2_. HIF2α mRNA expression after 24 hours of 2% O_2_ exposure was significantly higher in syncytiotrophoblast (Fig. 2C). These observations indicated that hypoxic responses of primary trophoblasts change across the syncytialization process from one dominated by HIF1α in cytotrophoblast cells to an HIF2α-dominant response in syncytiotrophoblast.

### HIF1α and HIF2α expression in villous human trophoblast

**Figure 3:**
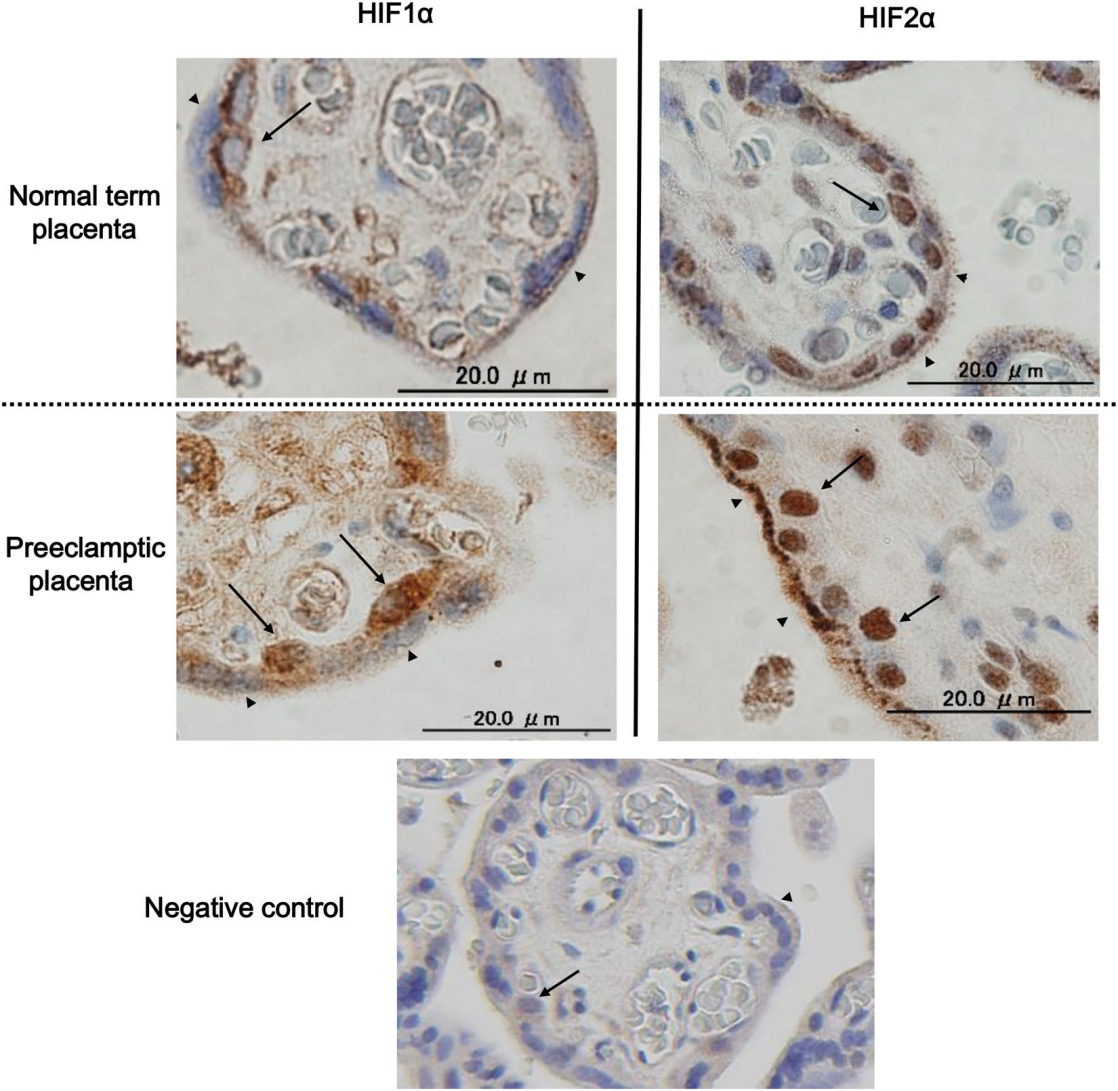
HIFα protein expression in normal and preeclamptic placentas. Immunohistochemistry was conducted to investigate the localization of HIF1α (figures in the left) and HIF2α proteins (figures in the right) in the villi of three normal term placentas (upper figures) and three preeclamptic placentas (lower figures). Clinical characteristics of the placenta examined were summarized in Table 1. The specific staining was visualized in brownish staining. Black arrows indicate cytotrophoblasts and arrow heads indicate syncytiotrophoblast. The representative result of normal term placenta (case #2 in Table 1), preeclamptic placenta (case #6 in Table 1) and negative control (case #6 in Table 1) are shown.

Immunohistochemical analysis was conducted to confirm the localization of HIF proteins in human placental tissues. HIF1α and HIF2α protein expression patterns in cytotrophoblast cells and syncytiotrophoblast were examined in three uncomplicated term placentas and three placentas complicated by early–onset PE. Representative images are shown in Fig. 3. Clear expression of HIF1α was confirmed in the cytotrophoblast cells residing at the inner surface of syncytiotrophoblast layer, but HIF1α-specific staining intensity was relatively dim in syncytiotrophoblast. Nuclear HIF2α was observed equally both in villous cytotrophoblasts and syncytiotrophoblast. These results support data from primary *in vitro* syncytialization experiments showing relatively enhanced expression of HIF2α compared to HIF1α in syncytiotrophoblast. These characteristics of HIF1α and HIF2α expression patterns were shared between uncomplicated and preeclamptic placentas. In comparison of staining intensities for HIF proteins, the difference between preeclampsia and uncomplicated pregnancy was not consistent among the placental samples examined.

### Impact of oxygen concentration on PlGF expression levels in primary trophoblast cells and trophoblast cell lines

**Figure 4:**
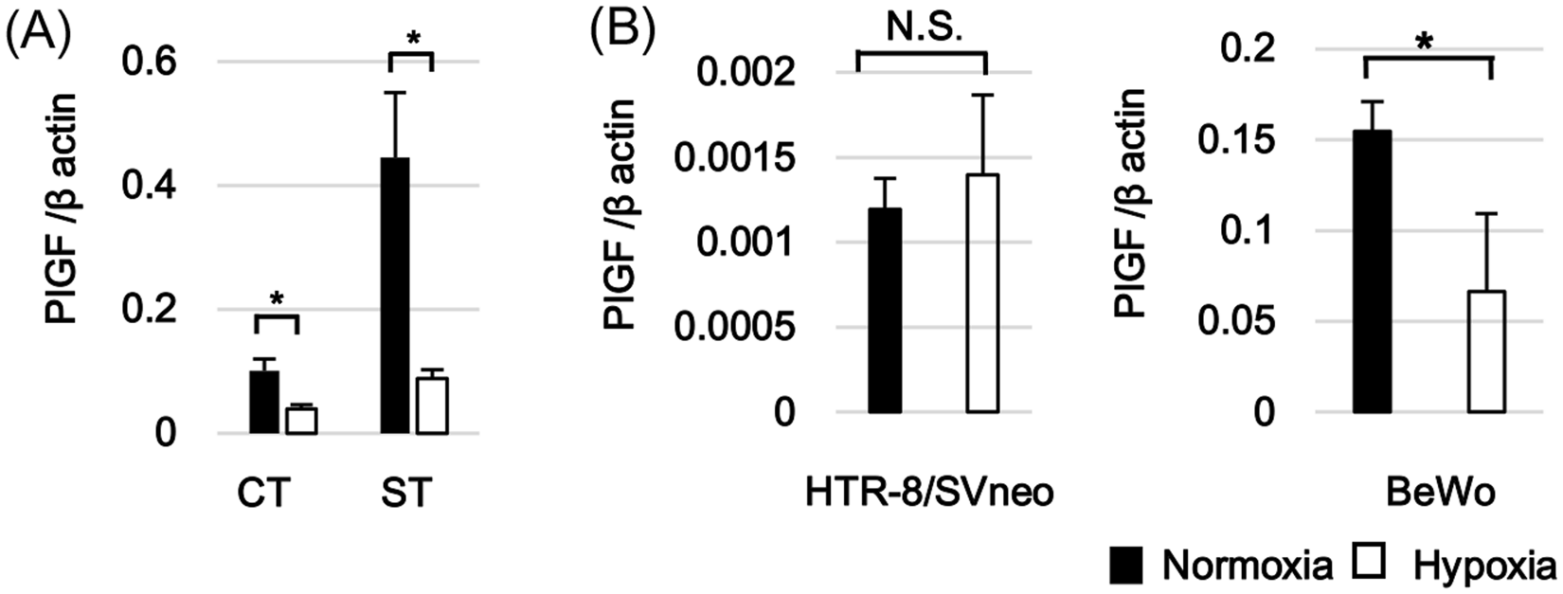
Alteration of placental growth factor (PlGF) gene expression in trophoblast cells in response to oxygen environment. (**A**) PlGF mRNA expression in primary trophoblast cells under different oxygen conditions. Freshly Isolated cytotrophoblasts (CT) and syncytiotrophoblast (ST) obtained by spontaneous differentiation of the cytotrophoblasts at 72 hours in culture were prepared. CT and ST were incubated under 20% O_2_ or 2% O_2_ conditions for 24 hours and PlGF mRNA expression was determined using real-time PCR. The data were obtained from eight independent experiments using cytotrophoblasts isolated from different placentas. (**B**) PlGF mRNA expression in trophoblast-derived cell lines under different oxygen conditions. HTR-8/SVneo and BeWo were incubated under 20% O_2_ or 2% O_2_ conditions for 24 hours. PlGF mRNA expression levels were determined using real-time PCR in the cells of each cell line. The data are based on at least three repetitive experiments. *p < 0.05, NS: not significant.

In this series of experiments, we examined whether trophoblasts PlGF mRNA expression levels were affected by oxygen levels in this culture. PlGF mRNA expression was evaluated using real-time PCR in the same syncytialization model described above. Freshly isolated cytotrophoblasts or syncytiotrophoblast formed 72 hours after seeding were exposed to 2% O_2_ or 20% O_2_ conditions for 24 hours (n = 8–9). Under 20% O_2_ conditions, PlGF mRNA expression levels were substantially higher in syncytiotrophoblast when compared with cytotrophoblast cells, linking PlGF expression to the process of *in vitro* syncytialization. PlGF mRNA expression was significantly suppressed in both cytotrophoblast cells and syncytiotrophoblast in response to 2% O_2_/hypoxic conditions. In summary, like HIF2α, PlGF transcription increased with syncytialization. Unlike the HIF proteins, PlGF transcription decreased under hypoxic conditions (Fig. 4A). We also examined PlGF responses to oxygen conditions in two common trophoblast cell lines, BeWo and HTR-8/SVneo. PlGF mRNA expression in BeWo and HTR-8/SVneo cells were evaluated following exposure to 2% O2 and 20% O2 for 24 hours. PlGF mRNA expression was significantly lower under 2% O_2_ than that under 20% O_2_ in BeWo cells but no change was noted in HTR-8/SVneo cells (Fig. 4B). The PlGF responsivity to hypoxia noted in primary trophoblast cultures was recognized in BeWo but not in HTR-8/SVneo cells.

### HIF1α and HIF2α exert distinct regulatory effects on PlGF expression

**Figure 5:**
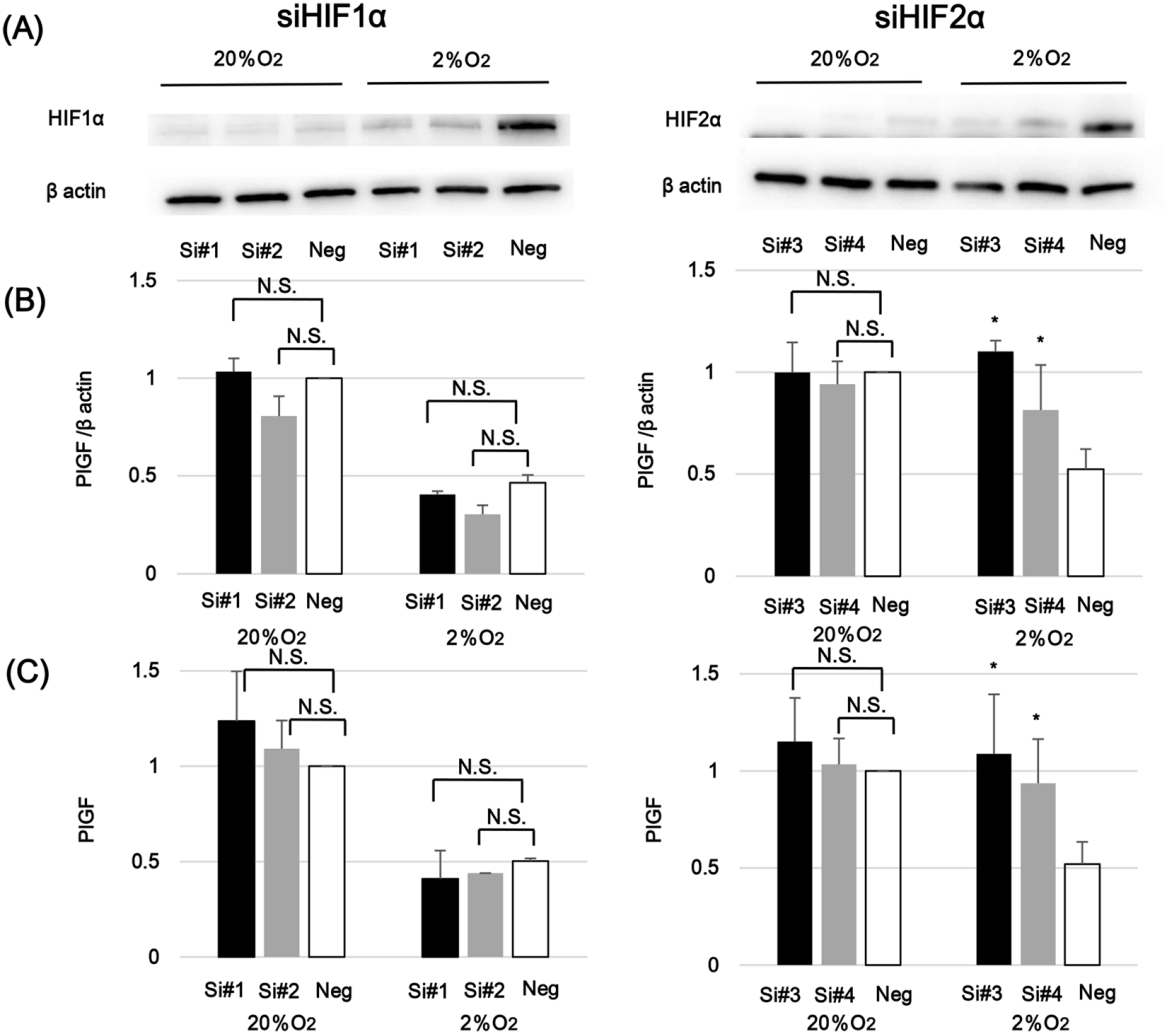
The involvement of HIFα isoforms in the regulation of PlGF production. Gene knockdown of HIFα isoforms were conducted in BeWo cells by transfection of siRNAs. BeWo cells were incubated under 20% O_2_ or 2% O_2_ conditions for 24 hours after HIF1α-specific siRNA (siHIF1α; Si#1 and Si#2) or HIF2-specific siRNA (siHIF2α; Si#3 and Si#4) was transfected. For the negative control, siRNA for non-specific target (Neg) was transfected. (**A**) Reduction of HIFα proteins by gene knockdown. Immediately after incubating for 24 hours under distinctive oxygen conditions following siRNA transfection, the protein amount of HIF1α and HIF2α in BeWo cells were evaluated by Western blotting analysis. β actin specific bands were detected for normalization of protein concentration variations among the samples. Cropped gels/blots around 100 kDa for HIFs and 45 kDa for β actin are displayed. (**B**) Impact of HIFα gene knockdown on PlGF mRNA expression. Immediately after incubating for 24 hours under distinctive oxygen conditions following siRNA transfection, PlGF mRNA expression levels were analyzed using semi-quantified real time PCR. In HIF1α (left) and HIF2α (right) silencing experiments, statistical analysis was performed between the negative control and each of two siRNAs in 20% O_2_ or 2% O_2_ conditions. The shown data are representative of at least three independent experiments. (**C**) Alteration of PlGF secretion by HIFα gene knockdown. At the same time point as (**B**), the culture supernatants of BeWo cells were collected and PlGF concentrations were measured by ELISA. In HIF1α (left) and HIF2α (right) silencing experiments, statistical analysis was performed between the negative control and each of two siRNAs in 20% O_2_ or 2% O_2_ conditions. The shown data are representative of at least three independent experiments. *p < 0.05.

We hypothesized that HIF proteins might be involved in the regulation of PlGF expression in trophoblast cells. To clarify this point, we separately suppressed HIF1α and HIF2α gene expression in BeWo cells using specific small interfering RNAs (siRNAs). Two pairs of siRNAs were prepared for HIF1α and for HIF2α. Since expression of HIF proteins in BeWo cells at baseline is low in 20% O_2_ conditions, no obvious effect of the siRNAs on HIF1α and HIF2α expression levels could be detected. (Fig. 5A). However, comparison with negative controls since the baseline expressions of target gene-specific suppression could be detected for both HIF1α and HIF2α under 2% O_2_ conditions.

We next used these siRNAs to assess the impact of HIF1α or HIF2α gene suppression on PlGF mRNA expression using real-time PCR. As expected, PlGF mRNA expression under 2% O_2_ was lower than under 20% O_2_ in BeWo cells transfected with negative control siRNAs. Both of the prepared HIF2α siRNAs (Si#3 and Si#4 in Fig. 5B) reversed this reduction in PlGF under hypoxia. PlGF expression levels following HIF2α siRNA transfections approached those under 20% O_2_ condition, particularly for Si#3. Suppression of HIF1α with either Si#1 or Si#2, did not affect hypoxia-induced PlGF suppression (Fig. 5B). Under 20% O_2_ condition, PlGF mRNA expression levels in HIF1α and HIF2α siRNA and negative control transfected BeWo cells were unaffected by treatment.

Finally, PlGF concentrations in culture supernatants of siRNA-transfected BeWo cells were also evaluated using ELISA. In agreement with mRNA expression results, HIF2α siRNAs reversed the suppression of PlGF secretion noted in response to hypoxic exposure. This alteration was not observed after transfection of HIF1α siRNAs (Fig. 5C).

## Discussion

In this study, we evaluated the protein and mRNA expression patterns of HIF1α and HIF 2α in an *in-vitro* syncytialization model in which highly purified cytotrophoblasts are differentiated into syncytiotrophoblast over the course of 96 hours. HIF2α expression increased with syncytialization while HIF1α expression decreased. Immunohistochemical analysis supported these *in-vitro* observations, showing intense HIF2α expression in syncytiotrophoblast and preferential HIF1α expression in villous cytotrophoblast cells. Finally, we demonstrated that HIF2α negatively regulates PlGF production in BeWo cells.

To our knowledge, this is the first report to evaluate the expression characteristics of HIF1α and HIF2α during the process of syncytialization. Although past immunohistochemical studies of human placenta have described the presence of HIF proteins in trophoblast cells^17–19^, none have specifically addressed expression patterns during trophoblast differentiation. Variations in HIF expression during cell differentiation have been reported in macrophage biology. In response to Th1 and Th2 cytokines, HIF1α and HIF2α mRNA is differentially expressed in M1- and M2-polarized macrophages^20^. HIF2α produced by macrophages in response to Th2 cytokines promotes arginase1 gene expression which is central to the development of the M2 phenotype^20^. Reflecting their restricted antigen presenting ability and suppressive effect on T-cell activities, M2 macrophages play a critical role in immune tolerance and immune evasion of cancer cells^21,22^. These properties are similarly important in the immunobiology of placental syncytiotrophoblast, the outer layer of fetal derived tissue that must avoid allogenic attack while bathed in maternal blood. Like M2 macrophages, syncytiotrophoblast exhibits HIF2α-biased expression responses. The molecular mechanism linking HIF2α activities to immunological regulation in syncytiotrophoblast could form the basis of future investigations on the role of the maternal immune system in normal and disordered human placentation.

PlGF is a VEGF family protein which is abundantly produced in the placenta, especially in syncytiotrophoblast^23^. PlGF can stimulate placental angiogenesis through binding to Flt-1^24^, although its angiogenic impact is relatively weak compared to VEGF. Regulation of PlGF production remains poorly understood. Ahmed *et al*., have shown that higher oxygen tensions are associated with enhanced PlGF protein production from term placental villous explants, whereas hypoxia decreases PlGF production in BeWo cells^25^. Consistent with their work, we confirmed in this study that PlGF expression was lower under hypoxic conditions when compared to ambient oxygen levels in both cultured primary syncytiotrophoblast and BeWo cells. In contrast, reduction of PlGF expression under hypoxia was not observed in HTR-8/SVneo cells. The ability for syncytialization is preserved in BeWo but not in HTR-8/SVneo. Considering that PlGF expression was highly upregulated following the syncytialization in primary trophoblasts, poor PlGF expression response to hypoxia in HTR-8/SVneo cells might be associated with their loss of ability for syncytialization. Additionally, we demonstrated that increases in trophoblast HIF2α expression lead to a down-regulation of PlGF gene expression under 2% O_2_ conditions. This is a novel link between HIF signaling and the control of PlGF production in the placenta. Since the knowledge on transcriptional regulation of PlGF gene is quite limited, the interaction of HIF2α with PlGF gene promoter is a subject for the future works. HIF1α-induced upregulation of VEGF is important to activation of angiogenesis by hypoxia^26^. PlGF is generally categorized as a proangiogenic factor, albeit a relatively weak one when compared to VEGF. Still, HIF2α-induced reductions in PlGF in response to low oxygen levels seem somewhat contradictory to the common biological phenomenon of angiogenesis in hypoxic conditions. It is possible that HIF2α-induced PlGF suppression and HIF1α-enhanced VEGF under lower oxygen condition might be particularly favorable for the rapid vascular development required for early placental development prior to unplugging of the uterine spiral arteries. In contrast, elevations in local oxygen concentrations following the establishment of inter villous flow should attenuate HIF1α protein activities, leading to diminished angiogenesis with a shift to a high PlGF/low VEGF balance.

In previous studies on HIFα isoform expression in preeclamptic placentas, HIF2α protein levels were clearly increased when compared to unaffected placentas^27,28^. Minimal changes were noted in HIF1α expression. Our study demonstrates that HIF2α rather than HIF1α is dominantly expressed under low oxygen conditions in syncytiotrophoblast. Additionally, our immunohistochemical study demonstrated that HIF2α was dominant in syncytiotrophoblast, although no obvious enhancement in HIF protein expression levels in preeclamptic placentas was confirmed. Taken together, HIF2α upregulation previously reported in preeclamptic placentas may reflect the response of syncytiotrophoblast to local ischemia and/or oxidative stress accumulated by ischemia-reperfusion damage as a consequence of spontaneous vascular constriction in incomplete uterine artery remodeling. Further, we provide new insight into the well-known phenomenon of reduced serum PlGF levels in preeclamptic women^29,30^. It is possible that enhanced HIF2α expression in preeclamptic placenta may lead to the decreases in placental PlGF secretion in PE patients. However, this speculation might be too simplistic, since there is a controversy over whether PlGF production is reduced in preeclamptic placenta. In some past studies, reduced PlGF mRNA expression was described in the placenta complicated with PE^31,32^. In contrast, recent systematic review and meta-analysis of the placenta denies diminished placental PlGF expression in PE patients^33^. Thus lower serum PlGF concentrations in the preeclamptic women is no more than the secondary phenomenon caused by increased placental sFlt-1 secretion which reduces unbound PlGF in the serum. On the other hand, it has been reported that in the first trimester of pregnancy, lower serum PlGF without remarkable increase in sFlt-1 in women developing PE in the later stage of gestation^34,35^. Taken together, it is possible that deranged PlGF expression resulting from increased HIF2α might be involved in the pathological event of preeclampsia in the early pregnancy.

The present study demonstrates that trophoblast cell differentiation is closely associated with the expression patterns of HIFα isoforms. In addition, HIF2α expression has a negative impact on PlGF production in syncytiotrophoblast. These findings suggest a novel relationship between HIFα protein activities and the VEGF system in human placenta.

## Methods

### Isolation of human villous cytotrophoblasts

This study is approved by the institutional review board of Faculty of Medicine, the University of Tokyo, and all experiments were performed in accordance with relevant guidelines and regulations (IRB number in our facility: 10979). After written informed consent was obtained, normal term placentas were collected from healthy pregnant women at 37 to 38 weeks of gestation at the time of selective cesarean section. We isolated villous cytotrophoblasts by modifying previously described protocols^36,37^. Briefly chorionic villous tissues were dissected away from the chorionic plate, basal plate and main vessels. Minced villous tissues were digested in 100 ml HBSS supplemented with 0.125% trypsin (Thermo Fisher Scientific, Waltham), 0.5 mg/ml DNase type1, 250 mg Dispase 2 (Sigma-Aldrich, St. Louis), 0.1 mM CaCl2 and 0.8 mM MgSO4 for 20 min at 37 °C. The cell suspension was collected and 5 ml FBS was added to inactivate trypsin. After passing through a 100 μm nylon filter, the collected cell suspension was layered over Percoll (GE Healthcare, Chicago) density gradients with four layers (50%, 45%, 25% and 20% Percoll layers). After the centrifugation, the floating cells between the 25 and 45% Percoll layerd were collected. Collected cells were incubated with an anti HLA-ABC antibody (Affymetrix, Santa Clara) followed by anti-mouse-IgG antibody-microbeads (Miltenyi Biotec, Bergisch Gladbach). Only HLA-ABC negative cells were collected using a Mini MACS TM separator (Miltenyi Biotec). Purified cytotrophoblasts were diluted in IMDM (GE Healthcare) supplemented with 10% fetal bovine serum (FBS), 1% Antibiotic-Antimycotic (Thermo Fisher Scientific), 200mM L-glutamine and 10ng/ml EGF and plated at a density of 1*10^6^ cells/ml on 6-well plates coated with collagen type 1. In the experiments designed to examine the impact of reduced oxygen environment, trophoblasts were exposed to 2% O_2_ condition (5% CO_2_/2% O_2_/93% N_2_) in a Bio Labo Multigas Incubator (Jujifield, Tokyo, Japan) from 72 to 96 hours post-plating.

### Cell lines

HTR-8/SVneo, a trophoblast-derived cell line, was kindly supplied by CH Graham^38^ and BeWo, a choriocarcinoma-derived cell line, was obtained from American type culture collection. HTR-8/SVneo cells were cultured in RPMI 1640 (Wako, Osaka) supplemented with 10% FBS, and BeWo cells in Ham’s F12 (Wako) supplemented with 10% FBS.

### Immunoassays for HCG and PlGF

HCG concentrations in culture supernatants were measured by chemiluminescence enzyme immunoassay using IMMULITE 2000 (Siemens Healthineers, Erlangen). This procedure was performed for fee by SRL, Inc. (Tokyo). PlGF concentrations in the culture supernatants were measured using a sandwich ELISA (Quantikine ELISA kit; R&D Systems, Minneapolis). Minimal detectable limits for these assays were 1.1 mIU/mL for HCG and 7 pg/ml for PlGF.

### Real time PCR

Total RNA from cultured cells were isolated using a Blood/Cultured Cell Total RNA Purification Mini Kit (FAVORGEN Biotech, Changzhi) according to the manufacturer’s instructions. Total RNA was reverse transcribed into cDNA and quantitative real-time PCR was performed using the Light Cycler thermal cycler system (Roche Diagnostics, Basel). The expression levels were normalized to β actin. The sequences of the oligonucleotides used as primers are listed below; HIF1α forward 5′-GAACGTCGAAAAGAAAAGTCTCG-3′, reverse 5′-CCTTATCAAGATGCGAACTCACA-3′, HIF2α forward 5′-GTGCTCCCACGGCCTGTA-3′, reverse 5′-CCTTATCAAGATGCGAACTCACA-3′, PlGF forward 5′-GTTCAGCCCATCCTGTGTCT-3′, reverse 5′-AACGTGCTGAGAGAACGTCA-3′, β actin forward 5′-CATGTACGTTGCTATCCAGGC-3′, reverse 5′-CTCCTTAATGTCACGCACGAT-3′.

### Western blot analysis

Protein was extracted from the cultured cells using the Cell Lytic™ MT Cell Lysis Reagent (Sigma-Aldrich) and Protease Inhibitor Cocktail (Nacalai Tesque,Kyoto). The extracted protein solutions were mixed with Sample Buffer Solution without Reducing Reagent (6x) for SDS-PAGE (Nacalai Tesque) and denatured for 5 min at 95 °C. 20 µg of the total protein samples were separated on Any kD™ Mini-PROTEAN® TGX™ Precast Protein Gels (BIO-RAD) and then transferred onto polyvinylidene difluoride membranes (EMD Millipore, Billerica). After a blocking with 5% skimmed milk in tris-bufferd saline with 0.1% Tween 20 for 2 hours at room temperature, the membranes were incubated with HIF1α (Novus Biologicals, Littleton), HIF2α (Abcam, Cambridge), or β actin (Cell Signaling Techonology, Danvers) primary antibodies overnight at 4 °C and secondary antibodies for one hour at room temperature. Chemiluminescence was visualized with ECL Plus (GE Healthcare).

### Immunohistochemistry

Three uncomplicated placentas were obtained from healthy pregnant women at 37 to 38 weeks of gestation at the time of selective cesarean section. Three preeclamptic placentas were collected in cesarean sections of the women with PE which was diagnosed at less than 34 weeks of gestation (early onset type). PE was defined hypertension emerged after 20 weeks of gestation accompanied with proteinuria (≧300 mg/day) or following any signs including impaired liver function, thrombocytopenia, new development of renal insufficiency, pulmonary edema and new-onset cerebral or visual disturbances according to the criteria defined in “Task Force Report on Hypertension in Pregnancy by the American College of Obstetricians and Gynecologists”. Clinical characteristics of the women providing the placental samples for immunohistochemistry are shown in Table 1 (#1, #2 and #3; normal control placentas #4, #5, #6; preeclamptic placentas) Collected placental villi were fixed in 4% paraformaldehyde phosphate (Wako) overnight at 4 °C. Samples were embedded in paraffin and sectioned at a thickness of 6 µm. After deparaffinization and rehydration, the sections were incubated with Peroxidase-Blocking Solution (DAKO, Carpinteria) for 10 min at room temperature. Antigen retrieval was performed using microwave exposure (Citric Acid, pH 6.0) for 15 min. Non-specific antibody binding was blocked by incubation in Protein Block, Serum-free (DAKO) for 2 hours at room temperature. The sections were incubated with 5 µg/ml of primary antibodies against HIF1α or HIF2α (Abcam) protein or non-immunized IgG (DAKO) for negative control overnight at 4 °C. After being washed in tris-bufferd saline, the sections were incubated with Envision Dual Link-HRP (DAKO) as a secondary antibody for 30 min at 37 °C and visualized using DAB substrate buffer (DAKO). Mayer’s haematoxylin stain was used as a counterstain.

**Table 1:** Clinical characteristics of the pregnant women providing the placental samples.

Case No.	Age	GW at delivery	Indication of CS	Birth weight (g)	Placental weight (g)	Umbilical artery pH value
#1	33	38w4d	Repeated CS	3512	720	7.19
#2	31	37w6d	Repeated CS	3070	680	7.29
#3	32	38w5d	Breech presentation	2688	455	7.26
#4	38	32w5d	Severe PE	1249	225	7.31.
#5	32	35w1d	Severe PE	1532	240	7.31
#6	33	32w4d	Severe PE	1422	310	7.34

GW: gestational week, CS: caesarean section.

### HIF expression silencing by small interfering RNA (siRNA)

Two pairs of HIF1α and HIF2α specific siRNA (si-HIF1α, si-HIF2α) and a non-specific siRNA (si-NC) were obtained from Thermo Fisher Scientific. BeWo cells were seeded on a six-well plate at a density of 30–40% confluence. At 12 hours after seeding, si-HIF1α, si-HIF2α or si-NC were transfected according to manufacturer’s instructions using lipofectamine (Thermo Fisher Scientific) and were further incubated for 24 hours. Culture media were changed and cells were cultured under 20% O_2_ or 2% O_2_ condition for additional 24 hours.

### Statistical analysis

Mann-Whitney U testing was conducted in the statistical analysis for mRNA levels in primary trophoblasts. Comparisons of multiple condition groups with controls were conducted using Dunnett’s testing. A p value of <0.05 was considered statistically significant. Data are shown as means ± SDs. All statistical analyses were performed using JMP PRO11 software (SAS Institute, Cary).
